# Isolated Congenital Lacrimal Gland Agenesis

**DOI:** 10.7759/cureus.57732

**Published:** 2024-04-06

**Authors:** Gokce Belge Bilgin, Cem Bilgin, Atakan Orscelik, Basel Musmar, Sedat G Kandemirli

**Affiliations:** 1 Department of Radiology, Mayo Clinic, Rochester, USA; 2 Department of Neurological Surgery, University of California San Francisco, San Francisco, USA; 3 Department of Neurological Surgery, Duke University, Durham, USA; 4 Department of Radiology, Boston Children's Hospital, Boston, USA

**Keywords:** ophtalmology, radiologic findings, pediatric patients, dry eye, lacrimal gland agenesis, congenital alacrima

## Abstract

Congenital alacrima is an uncommon condition marked by a lack of tear production that is present from birth. This condition often occurs in conjunction with various syndromes but can also result from isolated lacrimal gland agenesis. Congenital alacrima should be evaluated in the differential diagnosis for pediatric patients presenting with symptoms of dry eyes, especially in cases without xerostomia or other systemic rheumatologic findings. Following a thorough history and examination, noninvasive imaging techniques can be utilized to assess for potential lacrimal gland agenesis and aid in confirming the diagnosis.

## Introduction

Congenital alacrima is characterized by a lack of tear production present from birth. It is rare and usually associated with syndromes like anhidrotic ectodermal dysplasia and familial dysautonomia [1]. Additionally, it can be seen secondary to lacrimal gland agenesis [2,3]. Lacrimal gland agenesis is more commonly associated with additional findings like lacrimal punctum abnormalities and salivary gland aplasia in hereditary syndromes like Allgrove syndrome, blepharophimosis-ptosis-epicanthus inversus syndrome (BPES), lacrimo-auriculo-dento-digital (LADD) syndrome, and aplasia of the lacrimal and salivary glands (ALSG) syndrome [4-7]. However, lacrimal gland agenesis may manifest as an isolated finding with no family history present. Isolated lacrimal gland agenesis is an uncommon entity; however, it should be considered in the differential diagnosis in the pediatric age group presenting with dry eyes, especially in cases without xerostomia and other systemic rheumatologic findings. [8]. Prior to surgical intervention, noninvasive imaging modalities like magnetic resonance imaging (MRI) or computed tomography (CT) can be utilized to assess the possibility of lacrimal gland agenesis [9]. Herein, we describe the clinical and imaging features of two cases presenting with congenital alacrima with CT findings consistent with isolated lacrimal gland agenesis.

## Case presentation

Case 1

**Figure 1:**
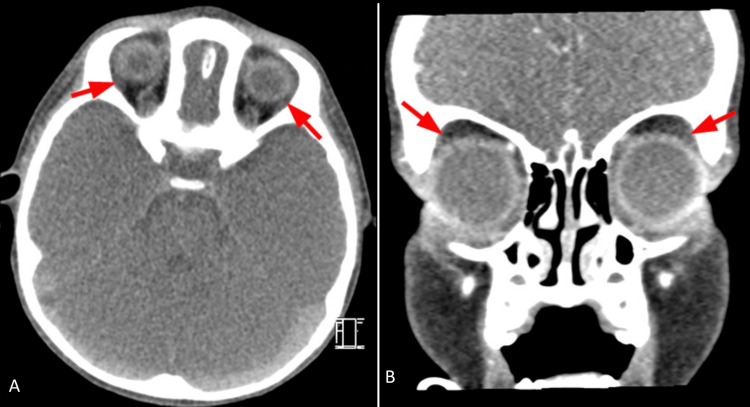
Congenital alacrima An 18-month-old patient presenting with congenital alacrima. Contrast-enhanced computed tomography (CT) images were obtained. Axial (A) and coronal (B) reformat images at the level of the lacrimal fossa show absence of the bilateral lacrimal glands (red arrows).

An 18-month-old patient was referred from an outside institution for further workup due to the absence of tears during crying noticed by parents after forearm subluxation. The patient was already using artificial tears at the time of presentation. The medical history was otherwise not significant. No family history of abnormalities related to sweating, salivation, arthritis, or neurological diseases was reported. Bilateral eye examination was unremarkable, with normal punctum and healthy corneal surface. Schirmer's testing and tear breakup time were not performed as the patient was already using artificial tears. Due to the immediate availability of CT imaging at our facility, a CT scan was ordered to assess the lacrimal glands. Contrast-enhanced CT of the orbits failed to show enhancing lacrimal glands at the lacrimal fossa (Figures 1A-1B). The major salivary glands were present and normal in size. Findings were consistent with isolated lacrimal gland agenesis. Continuing the symptomatic treatment with artificial tears was decided to protect the cornea. An additional anti-drainage procedure was suggested as part of the treatment plan, but the family opted to delay this procedure at that moment. The parents were educated about strategies to adapt to their surroundings to minimize the risk of increased evaporative loss from the ocular surface. On subsequent follow-up appointments, the patient did not experience any symptoms, so the symptomatic treatment with artificial tears was continued.

Case 2

**Figure 2:**
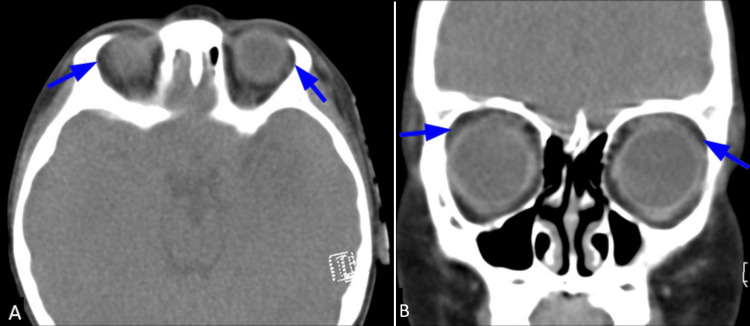
Congenital alacrima A four-year-old boy presenting with a foreign-body sensation and pain in both eyes. Contrast-enhanced computed tomography (CT) of orbits (A: axial; B: coronal sections) reveals bilateral absence of the lacrimal glands (blue arrows).

A four-year-old boy was referred for further workup when his parents noticed the absence of tears during crying. The patient was already prescribed artificial tears at the time of presentation; however, he was noncompliant with the regimen. He complained of a foreign-body sensation and pain in both eyes. During the examination, the visual acuity of both eyes was measured as 20/20. Slit-lamp examination revealed conjunctival hyperemia and corneal punctate epithelial erosions. Tear breakup time was less than three seconds in both eyes, and the patient was unable to tolerate Schirmer's testing. The medical history was otherwise insignificant, with no reported signs of xerostomia. A rheumatology panel yielded negative results. CT scan showed the absence of bilateral lacrimal glands, with normal major salivary glands (Figures 2A-2B). Since the patient's symptoms were present despite artificial tears, the bilateral punctal plug insertion was recommended. However, the parents expressed their preference to postpone the surgery. They were educated about the importance of treatment adherence and self-management techniques, including warm compresses, humidifier use, and minimizing exposure to wind and air conditioning. Subsequent follow-up appointments revealed a reduction in the patient's symptoms, indicating a positive response to consistent use of artificial tears.

## Discussion

Dry eyes can lead to corneal problems in children. A thorough physical examination and medical and family history with systemic workup are necessary to detect possible other findings of syndromic disorders [9]. Depending on the time of onset, the differential diagnosis includes rheumatologic diseases and congenital alacrima [10]. Congenital alacrima covers a spectrum of diseases, including the persistence of neonatal alacrima, familial dysautonomia, and lacrimal gland agenesis [1]. Agenesis of the lacrimal glands can be an isolated finding or sometimes associated with ectodermal defects in branchial arches, lacrimal punctum abnormalities, and major salivary gland agenesis [4-7,11]. Hereditary disorders affecting the lacrimal system are extensive. However, the main syndromes to be considered include Allgrove syndrome, BPES, LADD, and ALSG [4-7,11]. Lacrimal gland agenesis can also be an isolated finding with no family history; however, isolated lacrimal gland agenesis has been reported in a limited number of case reports in the literature [2,3,8,9]. 

During embryological development, lacrimal glands arise from the surface ectoderm at the superior conjunctival fornix [12]. The components of the lacrimal drainage system, including the canaliculi, lacrimal sac, and nasolacrimal duct, are also surface ectodermal in origin. Lacrimal gland development occurs during the second gestational month and requires an interaction between the epithelial and mesenchymal cells [12].

Although the lacrimal film is essential for maintaining a healthy ocular surface, some pediatric cases have been reported in the literature with no signs of keratitis [8,13]. This might be related to the physiology of tear production. Tear secretion has two components: a resting component by the accessory lacrimal glands and a reflex component by the main lacrimal glands [3]. The presence of accessory lacrimal glands may have prevented keratitis formation in these cases. Additionally, the lipid layer of the lacrimal film is thicker in infants, with slower evaporation and increased stability [8,13]. This increased stability of the lacrimal film, along with a lesser degree of ocular surface exposure in infants, may prevent drying of the ocular surface [8,13]. Beyond infancy, children may present with an ocular damage, as there are isolated lacrimal gland agenesis cases presenting with filamentary keratitis [2]. 

Lacrimal gland agenesis is more commonly associated with additional findings of salivary gland aplasia in hereditary syndromes. ALSG, an autosomal dominant disease with variable expressivity, is characterized by aplasia or hypoplasia of the lacrimal glands, lacrimal punctum, and major salivary glands (parotid, submandibular, and sublingual glands) [11,14]. LADD syndrome, an autosomal dominant disease, is characterized by aplasia or hypoplasia of the lacrimal glands and major salivary glands in addition to abnormalities affecting the face, ears, eyes, mouth, teeth, digits, and genitourinary system [15]. BPES is a craniofacial development disorder characterized by ptosis, blepharophimosis (narrowed horizontal palpebral fissures), telecanthus (an abnormally wide intercanthal distance), and epicanthus inversus (prominent fold of the skin from the medial aspect of the lower eyelid to the upper eyelid) [5]. There can be additional ocular abnormalities like lacrimal gland agenesis. Allgrove syndrome, also known as the 3A syndrome, is an autosomal recessive disease characterized by the triad of Addison's disease, achalasia, and alacrima [7]. Alacrima can be secondary to lacrimal gland hypoplasia or aplasia [7]. 

In the diagnostic approach to alacrima, Schirmer's test is utilized to objectively measure tear secretion [16]. The tear breakup time test is also employed to assess the stability of the tear film on the eye's surface. Following the identification of a reduced/lack of tear production, a comprehensive ophthalmic evaluation is an essential component of the diagnostic assessment. This evaluation may encompass examinations of the lacrimal system, eyelids, and blink reflex; assessment for potential complications in the cornea; and checks for visual acuity, the retina, and the optic nerve. Considering the potential association of congenital alacrima with systemic disorders, a thorough systemic examination and genetic counseling may also be incorporated into the evaluation. Imaging techniques such as orbital ultrasound, CT scans, or MRI are highly recommended for detecting any structural or developmental issues with the lacrimal glands.

Therapeutic options depend on the additional associated findings. Available management options include the instillation of artificial tears, surgical obliteration of the punctum, and tarsorrhaphy [3]. Self-management strategies are also available to reduce evaporative loss from the ocular surface. These include minimizing exposure to wind and air conditioning, avoiding extremely hot or cold weather conditions, enhancing air moisture using a humidifier, and utilizing a moisture chamber. Patients should be advised to return for further consultation if their symptoms persist despite adhering to their treatment regimen or if they experience the onset of new symptoms.

## Conclusions

Lacrimal gland agenesis should be considered as a possible diagnosis in pediatric cases exhibiting symptoms like dry eyes and conjunctival irritation. While patients might present with an isolated lack of tears, a thorough examination and history are necessary to evaluate for possible syndromic disorders. If additional systemic findings are present, patients should undergo thorough evaluations, including genetic testing, to guide families and promote proactive monitoring of their other children's health. Without other associated findings, physicians might prefer cross-sectional imaging or ultrasound imaging of the orbits to check for possible lacrimal gland agenesis before invasive interventions. Patients should be educated about potential exacerbating factors such as certain medications, prolonged screen time, exposure to air conditioning, and extreme weather conditions, as well as low-humidity environments, which may worsen symptoms and damage the corneal surface. Treatment with lubricant eye drops and anti-drainage procedures has been successful in managing most symptoms associated with lacrimal gland agenesis. Early detection of this condition is crucial to prevent permanent complications such as severe scarring, perforation, or blindness.
